# Hearing Loss and Cognitive Decline in the Aging Population: Emerging Perspectives in Audiology

**DOI:** 10.3390/audiolres14030040

**Published:** 2024-05-23

**Authors:** Naveen K. Nagaraj

**Affiliations:** Cognitive Hearing Science Lab, Communicative Disorders & Deaf Education, Utah State University, Logan, UT 84322, USA; naveen@usu.edu

**Keywords:** central auditory processing disorder, dementia, hearing loss, Alzheimer’s disease

## Abstract

In this perspective article, the author explores the connections between hearing loss, central auditory processing, and cognitive decline, offering insights into the complex dynamics at play. Drawing upon a range of studies, the relationship between age-related central auditory processing disorders and Alzheimer’s disease is discussed, with the aim of enhancing our understanding of these interconnected conditions. Highlighting the evolving significance of audiologists in the dual management of cognitive health and hearing impairments, the author focuses on their role in identifying early signs of cognitive impairment and evaluates various cognitive screening tools used in this context. The discussion extends to adaptations of hearing assessments for older adults, especially those diagnosed with dementia, and highlights the significance of objective auditory electrophysiological tests. These tests are presented as vital in assessing the influence of aging and Alzheimer’s disease on auditory processing capabilities and to signal cognitive dysfunction. The article underscores the critical role of audiologists in addressing the challenges faced by the aging population. The perspective calls for further research to improve diagnostic and therapeutic strategies in audiology, and emphasizes the need for a multidisciplinary approach in tackling the nexus of hearing loss, auditory processing, and cognitive decline.

## 1. Introduction

Hearing loss, as defined by the World Health Organization (2023), is the inability to hear sounds over 25 dBHL. In the United States (U.S.), it affects approximately 37.5 million adults, as reported by the U.S. Department of Health and Human Services (2021). Presbycusis (age-related hearing loss) is the most prevalent sensory deficit in the elderly. It affects a significant portion of the elderly population and is often linked to progressive sensorineural hearing loss [1]. Age-related hearing loss (ARHL) involves the degeneration of various auditory structures, including the mechano-transducing cochlear inner and outer hair cells, the stria vascularis, and the auditory nerve. This mixed pathology reflects both intrinsic cellular aging and cumulative extrinsic factors like noise exposure, ototoxic medications, lifestyle choices, health comorbidities, and genetic predisposition [2]. A significant recent development in our understanding of ARHL is its emergence as a modifiable risk factor for developing dementia. Studies have linked ARHL with cognitive decline, dementia, and Alzheimer’s disease. Possible explanations include the loss of auditory input due to hearing loss in individuals with ARHL, potentially leading to increased cognitive demands, social isolation, and changes in brain structure and function. Common pathological pathways like oxidative damage and inflammation are also involved [3,4,5]. Despite this, a causal link between ARHL and dementia is yet to be established definitively.

A growing body of research suggests a link between ARHL and changes in brain structure, specifically, decreased volumes in brain regions associated with auditory processing. These changes potentially predispose individuals to an increased risk of dementia. Notably, Lin and colleagues [6] observed decreased volumes in the primary auditory cortex using MRI in older adults with hearing loss, possibly resulting from reduced stimulation of this region due to degraded auditory input. This relationship between ARHL and brain volume reduction was also supported in the Baltimore Longitudinal Study of Aging [7], where participants with hearing loss exhibited an accelerated rate of brain volume decline over a 6.4-year period compared to their normal-hearing counterparts. Moreover, studies have highlighted the importance of brain regions such as the temporal lobe in cognitive impairment and early-stage Alzheimer’s disease [8], suggesting that volume losses in areas responsible for auditory processing may have broader implications for cognitive health. A 2022 scoping review by Slade and colleagues [9] suggests that ARHL causes anatomical and functional changes in the auditory cortex, such as decreased GABA (Gamma-aminobutyric acid) neurotransmitter levels and grey matter volume. ARHL also leads to increased activity in nonauditory brain regions as a compensatory mechanism.

According to a study published in the *Lancet* [7], hearing loss in the mid-life stage is identified as a modifiable risk factor that may significantly influence the development of conditions such as dementia later in life. This research highlights the importance of addressing hearing loss earlier, to potentially alter the trajectory of cognitive decline. A recent systematic review and meta-analysis [10] covering a comprehensive selection of 31 studies with 137,484 participants found notable cognitive benefits associated with hearing aid use. This analysis included 25 observational studies and 6 clinical trials. Of these, 19 studies—comprising 15 observational and 4 clinical trials—were included in quantitative analyses. The results demonstrated that using hearing devices in individuals with hearing loss was associated with a significant (19%) reduction in the risk of cognitive decline. Furthermore, a meta-analysis of eight of these studies, which involved 126,903 participants and had follow-up durations ranging from 2 to 25 years, highlighted a significantly lower cognitive decline among hearing aid users compared to those with untreated hearing loss. Additionally, an analysis of short-term effects in 11 studies with 568 participants, showed a 3% improvement in cognitive test scores. These findings emphasize the importance of hearing aids in potentially mitigating cognitive decline in individuals with hearing loss. In addition, the Aging and Cognitive Health Evaluation in Elders (ACHIEVE) study [11], a comprehensive randomized controlled trial, examined whether treating hearing loss in seniors can slow cognitive decline, a precursor to dementia. The researcher team enrolled 977 individuals aged 70–84 with untreated hearing loss in a three-year trial, dividing them into a hearing intervention group or a health education control group to assess the outcome on cognitive functions. The results showed no overall effect of hearing treatment in the combined cohort. However, detailed analyses revealed a significant 48% reduction in cognitive decline over three years in the higher Atherosclerosis Risk in Communities (ARIC) cohort, unlike in the lower-risk de novo cohort. This suggests that the benefits of hearing interventions to cognitive changes may vary across different population groups, and thus highlighting the potential importance of such interventions for older adults at increased risk of cognitive decline and dementia.

Cumulative research findings suggest that hearing intervention with the use of hearing aids has the potential to mitigate cognitive decline by decreasing the cognitive load from effortful listening and by preventing auditory deprivation, which may lead to structural brain changes. These findings imply that the role of audiologists has to evolve to encompass not only the management of ARHL but also the early detection and intervention of cognitive health issues. As the prevalence of cognitive impairment and dementia rises with the aging demographic, audiologists are more likely to encounter older clients with communication difficulties stemming from a combination of hearing loss and cognitive impairment.

## 2. Early Detection of Cognitive Impairment

Audiologists are in a unique position to observe early signs of cognitive decline during routine hearing assessments and hearing aid fitting appointments. Audiologists can educate patients and their families about the risks associated with untreated hearing loss, including its potential role in exacerbating social isolation [12], loneliness [5], depression [13], and cognitive decline [14]. In addition, evidence also suggests a link between hearing loss and frailty [15], indicating that older individuals with hearing loss are at a higher risk of frailty in later life regardless of various factors. Shen and colleagues emphasize the extended interaction time audiologists have with patients, often surpassing that of primary care physicians. The average duration of an office visit with primary care physicians is about 20 min, and with the audiologist, it is approximately 1.2 h. This extended interaction allows for detailed conversations that can reveal critical insights into a patient’s cognitive abilities, including memory issues and difficulties in spoken language comprehension and expression. The detection of such cognitive difficulties is crucial for referral for further medical evaluation and early intervention.

The responsibility of audiologists must extend beyond clinical duties to encompass patient education and counseling. Audiologists are uniquely positioned to inform patients and their families about the risks associated with untreated hearing loss, including its potential role in exacerbating social isolation, loneliness, depression, and cognitive decline. Furthermore, audiologists are instrumental in monitoring for signs of cognitive impairment. While they do not diagnose dementia, their role in referring patients for neurological or geriatric evaluation is vital in the context of observed cognitive concerns during hearing assessments [16,17,18]. Cognitive screening in audiology clinics, especially for older adults, is a growing area of importance. Audiologists, through extended patient interactions, can notice early signs of cognitive decline with appropriate cognitive screening tools. A variety of cognitive screening tools are available for the early identification of mild cognitive impairment or dementia in older adults. With training, these tools can be used by healthcare professionals, including audiologists, to gain insights into cognitive impairments.

Here is a summary of some of the available screening tools:

Mini-Cog™: This tool is specifically designed for early dementia detection in older adults. It is a quick and effective screening tool often used in various clinical settings. It involves Recall and the Clock drawing test as two main components. The simplicity and speed of the Mini-Cog test make it a popular choice for initial cognitive screening. It is especially useful because it does not require any special equipment and can be administered by healthcare professionals in a variety of environments (https://mini-cog.com/download-the-mini-cog-instrument/, accessed on 20 May 2024).

The Mini-Mental State Examination (MMSE): The MMSE includes a set of 11 questions that healthcare professionals use to assess cognitive function and screen for dementia. The MMSE takes approximately 5 to 10 min to administer and allows for a broad assessment of cognitive abilities, encompassing tasks from following basic instructions to performing elementary calculations. The availability of the MMSE in over 70 languages is particularly valuable, as it allows for accurate assessments in non-English-speaking populations. https://cgatoolkit.ca/Uploads/ContentDocuments/MMSE.pdf, accessed on 20 May 2024.

Modified Mini-Mental State Examination (3MS): 3MS is an extension of the MMSE, incorporating the original components and adding four new ones to broaden assessment areas. These additions include long-term memory, verbal fluency, abstract thinking, and an extra recall task, expanding the scoring range to 100 points. The 3MS, conducted through interviews, has shown a strong correlation between its telephone and in-person versions. It is useful for screening potential cognitive impairments or for conducting a quick cognitive check in clinical settings. https://adrc.usc.edu/3ms/, accessed on 20 May 2024.

The Alzheimer’s Disease Assessment Scale–Cognitive Subscale (ADAS-Cog)**:** This is a detailed test commonly used in research and clinical trials for evaluating cognitive functions. The ADAS-cog scale evaluates cognitive abilities across eleven tasks. These tasks include orientation, memory recall and recognition through word lists, comprehension and execution of instructions, language skills, and both ideational and constructional praxis, providing a comprehensive assessment of cognitive function. It is more comprehensive than the Mini-Mental State Exam, comprising 11 sections and requiring about 30 min to complete. https://www.fda.gov/media/122843/download, accessed on 20 May 2024.

The Addenbrooke’s Cognitive Examination Revised (ACE-R): ACR-R is a brief sensitive test that is also widely used for assessing cognitive functions and screening for various types of dementia, including Alzheimer’s disease. The ACE-R assesses key cognitive areas: attention and orientation, memory, verbal fluency, language, and visuospatial abilities. https://psycnet.apa.org/record/2006-21887-011, accessed on 20 May 2024.

The Montreal Cognitive Assessment (MoCA): MoCA is a widely used cognitive screening tool designed to assess various aspects of cognitive function. However, the MoCA is primarily a verbal assessment, involving spoken instructions and verbal responses. This can present challenges for individuals with hearing impairments, as they may have difficulty understanding or responding to the verbal components of the test. To address this, a non-auditory version of the MoCA has been developed, specifically adapted for individuals with hearing impairments. https://mocacognition.com/, accessed on 20 May 2024.

Rowland Universal Dementia Assessment Scale (RUDAS): RUDAS is a brief cognitive screening tool aimed at reducing the impact of cultural background and language differences on the evaluation of a person’s cognitive abilities. RUDAS is portable, requires minimal training for administration, and is freely available, making it suitable for use in various languages with the help of interpreters. It evaluates registration, visuospatial orientation, praxis, visuoconstructional drawing, judgment, memory recall, and language, thus offering a comprehensive tool for cognitive assessment in multi-ethnic communities. https://www.dementia.org.au/professionals/assessment-and-diagnosis-dementia/rowland-universal-dementia-assessment-scale-rudas, accessed on 20 May 2024.

The Frontal Assessment Battery (FAB): FAB is a concise tool designed for use in clinical settings to help distinguish between frontotemporal dementia and dementia of the Alzheimer’s type. It is particularly effective in patients with mild dementia (MMSE > 24). It evaluates six subdomains of frontal lobe function, requiring about 10 min to administer. https://psychscenehub.com/wp-content/uploads/2018/07/Frontal_FAB_Scale.pdf, accessed on 20 May 2024.

The Executive Interview (EXIT25) is a bedside screening tool designed to assess executive function deficits. It can be administered in about 15 min and does not necessarily require medical personnel for its application, as properly trained non-medical staff can also conduct the test. https://health.utah.edu/sites/g/files/zrelqx131/files/files/migration/image/exit25.pdf, accessed on 20 May 2024.

The Kimberley Indigenous Cognitive Assessment (KICA): KICA is the sole validated tool for dementia assessment in older Indigenous Australians, which was developed and validated in Western Australia’s Kimberley region and the Northern Territory. It is tailored for rural and remote Indigenous populations, with a short version (KICA-Screen) validated in Far North Queensland. Created with input from local Indigenous communities, language centers, and health professionals, KICA is recommended for individuals aged 45 years and above in rural and remote areas where other dementia assessments may not be suitable. https://www.dementia.org.au/professionals/assessment-and-diagnosis-dementia/kimberley-indigenous-cognitive-assessment-tool-kica, accessed on 20 May 2024.

Additionally, there are tools designed for use by informants (family members and close friends) which include:

Eight-item Informant Interview to Differentiate Aging and Dementia (AD8): This tool is designed for informants to help differentiate between normal aging and potential signs of dementia. https://alz.org/media/documents/ad8-dementia-screening.pdf, accessed on 20 May 2024.

Short Informant Questionnaire on Cognitive Decline in the Elderly (IQCODE): This questionnaire is available in multiple languages and is used by informants to assess cognitive decline in the elderly. https://www.alz.org/media/documents/short-form-informant-questionnaire-decline.pdf, accessed on 20 May 2024.

General Practitioner Assessment of Cognition (GPCOG): This is specifically designed for use by general practitioners, primary care physicians, and family doctors to screen for dementia in a primary care setting. GPCOG is a brief tool used for screening dementia, consisting of an evaluation of the patient and an interview with a caregiver. It is designed to be completed in approximately 4 to 6 min. https://gpcog.com.au, accessed on 20 May 2024.

These tools are useful for detecting early signs of cognitive decline and assisting in timely interventions and care planning for older adults. However, differentiating cognitive issues from hearing impairments presents challenges as symptoms often overlap. Symptoms like trouble following conversations and avoiding social situations are common indicators of both auditory and cognitive impairments [19]. Standard cognitive screening tools may not be fully effective for those with hearing impairment, necessitating adapted or alternative tools specifically developed for adults with hearing loss such as the Montreal Cognitive Assessment: Hearing impairment version (MoCA-HI): MoCA-HI is a validated, sensitive, and reliable cognitive screening test for people with hearing impairment.

The risks associated with cognitive screening by audiologists include potential misdiagnoses due to overlapping symptoms, reliance on screening tools not designed for patients with hearing loss, and the interpretation of results without adequate training. There is also a risk of misattributing cognitive symptoms to hearing loss alone, which could delay or overlook necessary interventions for cognitive issues. Therefore, while cognitive screening in an audiology clinic is valuable, it requires appropriate training, careful implementation, and an understanding of its limitations to ensure effective and accurate patient care. 

Hence, collaboration with other healthcare professionals is a crucial aspect of the audiologist’s role. A multidisciplinary approach, involving primary care physicians, neurologists, and geriatric specialists, ensures comprehensive care for patients with ARHL, especially considering the cognitive dimensions of hearing loss. The expertise of audiologists in identifying early cognitive decline is essential for providing comprehensive healthcare for the aging population. This highlights their important role in the clinical management of patients and in wider public health initiatives.

## 3. Hearing Assessment Considerations

Regular hearing testing, particularly for the middle-aged and older individuals, is important for timely intervention for hearing loss. This proactive approach is crucial, as evidenced by recent findings from the ACHIEVE study, which revealed that hearing intervention has the potential to reduce cognitive decline [11]. People with dementia often have difficulties with attention, memory, and language comprehension, making standard auditory tests challenging to complete. Behavioral pure-tone audiometry and speech audiometry are standard initial tests in audiology clinics for assessing hearing in adults. However, these tests can be difficult for adults with dementia due to cognitive decline, thereby affecting the reliability of test results. In a study by Burkhalter et al. [20], an examination of medical records for 307 adults (in care facilities) exhibiting dementia symptoms revealed that only 32% were able to reliably complete pure-tone audiometry. Although not exclusively focused on confirmed dementia cases, this finding brings into question the effectiveness of pure-tone audiometry for adults with cognitive impairment. In a systematic review [21], it was reported that between 41% and 43% of adults with dementia were unable to complete pure-tone audiometry. This necessitates either adapting current procedures or employing alternative diagnostic tests for this demographic.

The creation of reliable hearing assessments is possible by modifying standard audiological testing procedures to accommodate the unique challenges faced by clients with dementia [22]. Adapting hearing assessments for the elderly with dementia involves various strategies by audiologists. Key approaches may include involving family and caregivers, conducting assessments in familiar environments, simplifying communication, and adjusting test procedures to suit the individual’s cognitive abilities. Strategies such as using pulsed tones, reducing test duration, and focusing on fewer frequencies can enhance the accuracy of audiometric information. Accommodating the patient’s comfort by allowing familiar faces during testing and using verbal responses instead of button presses is recommended. If standard methods fail, using objective tests like auditory evoked potentials or otoacoustic emission must be considered. These tests help in assessing hearing abilities even when standard behavioral tests may not be feasible due to the cognitive limitations associated with dementia.

## 4. Central Auditory Processing Considerations

In addition to hearing loss, central auditory processing disorders (CAPDs) have been implicated in association with Alzheimer’s disease, and they potentially manifest prior to the emergence of Alzheimer’s dementia. Consequently, CAPD represents a potential biomarker for the early detection of Alzheimer’s disease [23,24,25]. CAPD involves difficulty in perceiving sounds and understanding speech, unrelated to peripheral hearing loss, but rather due to deficits in the brain’s ability to process auditory information. Studies have consistently demonstrated a significant association between age-related CAPD and both MCI and dementia. This relationship suggests the involvement of central auditory pathways in the neurodegenerative process associated with these cognitive disorders [26,27].

In a seminal study, Sinha et al. [27] investigated the patterns of auditory system degeneration in patients with Alzheimer’s dementia. They observed a specific and consistent degeneration in the auditory system, characterized by the presence of senile plaques and neurofibrillary tangles in the ventral nucleus of the medial geniculate body and the central nucleus of the inferior colliculus in all nine patients with Alzheimer’s who were examined. Notably, adjacent nuclei within the medial geniculate body and inferior colliculus were unaffected. These pathological markers were also found in the primary auditory and auditory association cortices. In contrast, control tissues showed no senile plaques and neurofibrillary tangles in these regions, and the cochlear nuclei were normal in both Alzheimer’s disease and control patients. The study highlights that the ventral nucleus of the medial geniculate body, a major thalamic relay for auditory processing, and the inferior colliculus are particularly affected in Alzheimer’s disease, suggesting a potential loss of neurons across all frequency ranges. This contrasts with presbycusis in the elderly, which typically involves high-frequency loss due to peripheral lesions. The findings suggest that the observed histological changes in the brains of patients with Alzheimer’s disease could contribute to altered cognitive functions due to primary sensory deafferentation, thus offering new insights into the neurodegenerative processes of Alzheimer’s disease. However, distinguishing between normal cognitive decline associated with aging and the pathological dysfunction seen in the early stages of Alzheimer’s disease is challenging due to the subtle onset of the disease. The intricate relationship between auditory perception difficulties and speech communication issues in age-related CAPD is challenging to establish due to the concurrent presence of peripheral ARHL and cognitive changes in older individuals. This complexity underscores the challenge of delineating direct causal relationships between these disorders [5,26].

CAPD is typically suspected in individuals who struggle to understand speech in noisy environments, a problem that is common among the elderly [28]. While most people with age-related CAPD can communicate effectively in quiet situations, they struggle in noisy situations, a phenomenon often referred to as the “cocktail party effect”, “central presbycusis”, or “age-related processing disorder” [28,29]. The underlying mechanisms of CAPD are not completely understood, but dichotic listening tests have been extensively used to explore interhemispheric interactions and callosal functions [30]. Early studies [31] have linked the diminished ability to divide attention into dichotic tasks in patients with Alzheimer’s disease to anterior temporal lobe degeneration and reduced glucose metabolism. Further research points to the involvement of the parietal and frontal lobes, affecting attention and various executive functions, including the planning and initiation of activities [32]. CAPD testing, which involves discerning auditory signals in noise or among competing signals, demands significant attention and processing resources. Gates and colleagues [25] studied a cohort of 274 volunteers who underwent auditory testing and were followed for up to four years. The study found that lower scores on CAPD tests were significantly associated with a higher risk of developing dementia. Elderly individuals who exhibit significantly poor performance (scores below 50%) in dichotic competing speech tests, and yet maintain normal or near-normal speech recognition in quiet environments, might be experiencing cognitive decline.

Tuwaig and colleagues [33] evaluated two CAPD tests as potential markers for early Alzheimer’s disease progression in older individuals at increased risk. These were the Synthetic Sentence with Ipsilateral Competing Message (SSI-SCM) and Dichotic Sentence Identification (DSI) tests which are thought to assess distinct auditory capabilities. Specifically, the right-ear advantage in the DSI test is dependent on interhemispheric communication and integrity. Elevated scores on the DSI-right-ear advantage test, indicative of functional decline, were associated with cortical thinning in multiple areas. These areas included the left superior and transverse temporal gyri, both inferior temporal gyri bilaterally, the right anterior temporal pole, the precuneus, as well as the dorsomedial and inferior frontal gyri. In line with prior research [34], whole-brain analysis revealed a significant correlation at the peak level between SSI-ICM scores and the cortical thickness of the Heschl’s gyrus. Additionally, the SSI-ICM scores were associated with the cortical thickness in the inferior parietal lobule, a region involved in sensory integration, inclusive of auditory information. This area is known to exhibit significant atrophy and neurodegeneration in Alzheimer’s disease [35]. Notably, they also uncovered a previously unidentified link between SSI-ICM scores and the thickness of the para-hippocampal gyrus and entorhinal cortex. These regions are particularly susceptible to early atrophy in the pre-symptomatic stages of Alzheimer’s disease [36].

Overall, findings suggest that CAPD testing might be a useful tool for the early identification of cognitive disorders such as Alzheimer’s dementia, particularly in older adults who may be experiencing difficulty hearing in noise. An important direction for future research involves conducting a larger-scale study to assess the prevalence and long-term outcomes of this phenomenon, with the ultimate aim of developing targeted intervention strategies for this demographic. Patients who score exceptionally low (below 50% accuracy) on CAPD tests, yet have no known diagnosis of dementia, should be prioritized for further cognitive function evaluation and referral. Based on current evidence, it is also important to consider referring such cases for neurological evaluation. 

Despite the challenges in establishing causality, age-related CAPD has emerged as a potential diagnostic marker for cognitive dysfunction in the elderly. This marker indicates a specific link to neurodegenerative processes [26,37]. The primary clinical method for identifying age-related CAPD involves auditory behavioral assessments. Historically, CAPD tests have primarily been validated in patients with specific and well-defined lesions such as tumors in regions associated with certain auditory functions, such as the brainstem or temporal lobe. A recent meta-analysis [38] examined central auditory processing functions in Alzheimer’s disease and its preclinical stages through behavioral tests. It found that individuals with MCI significantly underperformed compared to healthy controls in several auditory processing tests. Similarly, participants with Alzheimer’s dementia showed poorer performance in Dichotic Digits, DSI, and SSI-ICM tests. These results highlight the potential of using auditory processing tests to identify cognitive decline stages. Considering the wide range of tests employed in audiological practice for defining age-related CAPD, tests that are most frequently utilized in epidemiological studies to examine the correlation with cognitive impairment are summarized below.

### 4.1. Speech in Noise Tests

The ability to understand speech in noisy environments declines with age, presenting more significant challenges for the elderly compared to younger adults. This phenomenon has been documented in various studies [25,39,40,41], emphasizing the role of auditory processing and cognitive functions in speech perception in noise. The anatomical pathways critical for distinguishing meaningful signals from noise are particularly governed by areas responsible for attentive executive functions, such as the dorsolateral prefrontal cortex. The prefrontal cortex primarily controls executive functions and working memory, which are particularly important when individuals process rapid auditory inputs to comprehend the intended meaning [42,43]. Speech perception becomes challenging and there is increased cognitive effort when the auditory signal is degraded by the presence of noise. This additional effort can interfere with other cognitive-linguistic processes. Working memory plays a crucial role here: if an incoming auditory signal is degraded, it needs to be retained for a longer duration, allowing additional cognitive systems more time to process it effectively. In a large-scale study [44] involving around half a million participants aged 40 to 60 years, it was found that speech perception in noisy environments declines exponentially after the age of 50, with a more dramatic decline in those with lower cognitive scores. This study also noted that cognitive decline and aging independently affect speech perception in noise ability. Additionally, men reported more hearing difficulties than women, and exposure to workplace noise was linked to both subjective and objective hearing challenges. The findings suggested that older people’s reduced ability to hear speech in noise is associated with declining cognitive processing and greater subjective hearing difficulty.

The Quick Speech in Noise (QuickSIN) test is one of the most common speech-in-noise tests used in clinics; moreover, Wong et al. [45] found that older adults differ significantly at the QuickSIN signal-to-noise level of 0 dB. Hence, we could argue that the QuickSIN test could be a useful screener for possible CAPD and decreased cognitive function when the performance is drastically worse than expected.

### 4.2. Dichotic Listening Tests

Dichotic listening involves simultaneously presenting different auditory signals to each ear. Dichotic listening tests, such as the Dichotic Digits and DSI test, are increasingly recognized as potential biomarkers for pre-clinical Alzheimer’s disease. Research [33] studies have found a strong relationship between CAPD and the subsequent incidence of Alzheimer’s, particularly through the DSI test, which showed a significant hazard ratio of 9.9. The results from this test, along with other CAPD tests like the SSI-ICM, indicate that different auditory processes reflect various aspects of cognitive decline. Specifically, while the SSI-ICM correlated with cortical thickness, the DSI was sensitive across multiple cognitive modalities and was linked with pathological changes and volumetric and cortical measures, highlighting its potential as a robust biomarker for Alzheimer’s disease.

Systematic reviews and meta-analyses further highlight the utility of Dichotic Digits in detecting cognitive impairments linked to Alzheimer’s disease [46,47]. In comparing patients with dementia and cognitively healthy controls [46], the findings demonstrated that individuals with dementia had significantly lower Dichotic Digits scores, with an average difference of 18.6%. Moreover, those with dementia exhibited a pronounced right-ear advantage, which was 24.4% greater compared to controls. These outcomes suggest that a decline in Dichotic Digits performance and an increase in right-ear advantage are potentially linked to the progression of cognitive impairment.

The research by Bouma and Gootjes [47] utilized Kimura’s Dichotic Digits paradigm to assess left hemispheric dominance for language processing in the elderly and patients with Alzheimer’s. The study highlighted that structural brain changes and attentional mechanisms significantly influence auditory processing, particularly ear asymmetry in dichotic listening tasks. Elderly participants, and more so those with Alzheimer’s, had notable difficulties focusing on the left ear, leading to a right-ear advantage. These challenges were linked to a breakdown in the cortical attentional network, involving both frontal regions (responsible for inhibitory control of attention) and parietal regions (associated with spatial attention). In addition, both interhemispheric (e.g., callosal atrophy) and intrahemispheric (e.g., subcortical white matter lesions) dysconnectivity were identified as significant factors contributing to these auditory processing challenges in patients with Alzheimer’s dementia.

Research across both cross-sectional and longitudinal studies has highlighted the influence of demographic and cognitive factors on Dichotic Digits performance. A comprehensive cross-sectional study [48] revealed that age, gender, education level, hearing loss severity, and cognitive impairment significantly influenced Dichotic Digits performance, accounting for 22.7% of the variability in scores. This highlights the complexity of Dichotic Digits test outcomes and suggests that, while the Dichotic Digits test is a valuable tool for assessing central auditory processing, its efficacy can be affected by a variety of demographic and cognitive factors. Moreover, a longitudinal study [49] showed a decline in free recall performance over a five-year period, while right-ear advantage tended to increase with age. These findings indicate that different components of the Dichotics Digits test may reflect distinct aspects of aging and cognitive function. Thus, it is essential to consider these demographic and cognitive factors when interpreting dichotic test results, as they significantly contribute to variability in test outcomes and may influence diagnostic accuracy in clinical settings.

### 4.3. Temporal Processing Test

Temporal processing in the auditory system is the ability to perceive and differentiate brief changes in the duration of sound stimuli. This aspect of hearing is essential for recognizing timing differences in speech, particularly in environments with background noise or multiple sound sources [50,51]. A key aspect of temporal processing involves detecting gaps within continuous auditory signals, whether they are noise or distinct sounds [52,53]. Various tests have been developed to evaluate temporal processing capability in adults. However, temporal processing is not solely an auditory phenomenon; it is closely interlinked with cognitive functions such as working memory and executive attention. These cognitive aspects, which tend to decline with age, significantly impact the ability to understand words, independent of the individual’s hearing status [54,55]. Consequently, assessing temporal processing, especially in the context of aging and cognitive impairment, requires a nuanced approach that accounts for both auditory and cognitive factors.

### 4.4. Auditory Electrophysiological Tests

Electrophysiological tests have been instrumental in providing objective assessments of central auditory processing, enhancing our understanding, and supplementing behavioral observations. These assessments have been particularly valuable in exploring age-related anatomical changes that affect central auditory processing. Notably, significant research has focused on the influence of aging on the auditory brainstem response (ABR), middle latency response (MLR), the late latency response (LLR), and the P300 event-related potential (ERP). These studies have revealed important insights into how aging influences auditory processing at both the structural and functional levels.

A recent meta-analysis has indicated significant delays in the ABR wave V, as well as in the interpeak intervals I–V and I–III, in patients with Alzheimer’s disease compared to controls [56]. ABRs, with their multiple neural generators along the brainstem auditory pathway, are commonly used to assess the functionality and integrity of both central and peripheral auditory pathways. These findings align with the hypothesis of brainstem and midbrain structural abnormalities in patients with Alzheimer’s disease. Variability in Alzheimer’s disease severity and duration might explain the discrepancies in ABR results across studies; however, overall, the pooled data from this meta-analysis support the presence of notable ABR abnormalities in Alzheimer’s disease compared to controls.

In addition, late latency auditory ERPs such as P50, N100, P200, N200, and P300 also revealed significant abnormalities in patients with Alzheimer’s disease compared to controls. Particularly, significant delays in N100, P200, N200, and P300 latencies were observed when using an active two-tone oddball paradigm. In the mild stage of Alzheimer’s disease, the most extensively studied changes in ERP pertain to the P300b or classic P300 component. This ERP component, triggered by a task-related deviant stimulus, is indicative of working memory updates. P300b amplitude is influenced by the allocation of attentional resources during the updating of working memory [57]. The latency of P300b, on the other hand, mirrors the speed of stimulus evaluation and classification. Studies comparing patients with Alzheimer’s disease and healthy controls typically report reduced P300b amplitude and prolonged latency in patients with Alzheimer’s disease. This pattern is especially pronounced in easy auditory oddball tasks, where discrepancies in the P300b amplitude are more significant than latency differences. In patients with mild Alzheimer’s disease, the N100 amplitude, similar to the P300, was significantly decreased [58].

Although N100 primarily reflects stimulus characteristics, it is also influenced by attention and memory factors. The reduced N100 amplitude in mild Alzheimer’s disease might be a manifestation of attention and memory deficits [59]. Despite sensory cortices typically being unaffected until the later stages of Alzheimer’s disease, a decrease in N100 amplitude may indicate alterations in inputs from brain regions associated with higher cognitive processes, which are more directly impacted in the early stages of the disease. These changes could involve regulatory inputs from areas like the prefrontal cortex and nucleus basalis, known to modulate auditory cortical responses. The N200, a negative peak preceding the P3b in ERP, is integral to cognitive processes like stimulus identification and distinction [60]. Its peak latency correlates with executive function and attention measures [61]. Studies have found delayed latency and reduced amplitude for the N200 in patients with Alzheimer’s Disease [61]. N200 latency is particularly effective in distinguishing patients with Alzheimer’s disease from those with MCI and healthy controls, while N200 amplitude, combined with P300 latency, helps monitor cognitive function changes over time in MCI [61,62]. These findings suggest that both latency and amplitude of N200 are affected in AD. The P300 amplitude was notably smaller in participants with Alzheimer’s disease, signifying its potential role in cognitive processes like attention, memory, and executive functions.

As highlighted above, significant variability in the ABR and late latency auditory ERPs such as P50, N100, P200, N200, and P300 has been documented across studies of patients with Alzheimer’s Dementia [56]. The effects of Alzheimer’s disease on ERPs can vary depending on several factors including the specific components of the ERP being measured, the stage of the disease, age, degree of hearing loss, and individual differences among patients. Research shows that, while some ERP components may exhibit delays or amplitude reductions in individuals with Alzheimer’s, others might not show significant changes. The variability in findings can be attributed to the complexity of the disease and how it affects neural pathways involved in auditory processing.

Although there is a well-documented association between hearing loss and cognitive impairment in the literature, differentiating the specific contributions of hearing loss from those of cognitive impairment on auditory ERPs poses a significant challenge. The difficulty arises because both hearing loss and cognitive decline can independently or synergistically affect the neural processes underlying these evoked responses. For example, hearing loss may primarily impact the earlier sensory components of ERPs due to degraded auditory input, whereas cognitive decline might more significantly affect later components associated with memory and attention processes. This study [63] indicated that age affects the N1-P2 component, but not MMN. This suggested that the later cognitive processing stages of stimulus discrimination, reflected in MMN, may be more susceptible to the effects of peripheral hearing loss on processing speech in noise than earlier sensory encoding stages signaled by N1-P2. Furthermore, the significant effects of hearing loss were observed both on MMN and on behavioral measures of speech perception.

There are significant implications for the use of ERPs as a potential clinical tool in assessing an individual’s capacity to perceive speech in challenging auditory environments. Current findings highlight the necessity for methodologies that can better differentiate these effects, and future studies could employ advanced ERP paradigms that control for hearing acuity or utilize simulation techniques to model the specific influence of hearing loss on ERP measures. In addition, integrating hearing aids or assistive listening devices during ERP testing could provide insights into how auditory amplification affects cognitive processing signals in both healthy aging populations and those with Alzheimer’s Dementia. Such approaches will be crucial for developing a nuanced understanding of how central auditory processing deficits interact with peripheral hearing loss to affect cognitive functions in aging and neurodegenerative diseases. Continuing to refine these methods will also facilitate the creation of diagnostic and intervention strategies that are sensitive to the early detection of dementia, considering individual variances in test performance and the combined effects of sensory and cognitive impairments in a comprehensive CAPD test battery., Future research should focus on creating a CAPD battery that is sensitive to the early detection of dementia, considering the variance in individual test performance. There is a significant need for future longitudinal studies with larger sample sizes and robust assessment tools. Such research is pivotal for disentangling cognitive dysfunction from sensory impairments and enhancing our understanding of the association between age-related CAPD and cognitive disorders. In summary, while a robust association between age-related CAPD and cognitive decline, including MCI and dementia, is evident, further research is warranted to elucidate the direction of this relationship and to develop efficacious diagnostic and intervention strategies.

## 5. Conclusions

In conclusion, in this perspective I elucidate the complex relationship between age-related CAPD, and cognitive decline associated with Alzheimer’s disease, underscoring the critical role of audiologists. Based on extant evidence, I discuss the necessity of refining diagnostic and management strategies for individuals with hearing impairments alongside the early detection of cognitive health issues and emphasize the need to adapt clinical hearing testing methods for the elderly, especially those with dementia. The incorporation of auditory electrophysiological tests by audiologists could provide new insights into how aging and Alzheimer’s disease affect auditory processing. Moreover, CAPD testing could emerge as a significant diagnostic marker for cognitive dysfunction, representing a notable clinical advance. The ultimate aim of this perspective is to highlight the essential role of audiologists in a multidisciplinary approach to meet the challenges of hearing loss, auditory processing, and cognitive decline in the aging population. The insights presented on CAPD testing are pivotal for enhancing our understanding of these complex interrelations and act as a springboard for future research and improvements in clinical practice.
